# Emergence of the novel SARS-CoV-2 lineage VUI-NP13L and massive spread of P.2 in South Brazil

**DOI:** 10.1080/22221751.2021.1949948

**Published:** 2021-07-15

**Authors:** Fernando Hayashi Sant’Anna, Ana Paula Muterle Varela, Janira Prichula, Juliana Comerlato, Carolina Baldisserotto Comerlato, Vinicius Serafini Roglio, Gerson Fernando Mendes Pereira, Flávia Moreno, Adriana Seixas, Eliana Márcia Wendland

**Affiliations:** a Hospital Moinhos de Vento, PROADI – SUS, Porto Alegre, Brazil; b Graduate Program in Biosciences, Federal University of Health Sciences of Porto Alegre (UFCSPA), Porto Alegre, Brazil; c Department of Chronic Conditions and Sexually Transmitted Infections, Ministry of Health, Brasília, Brazil; d Department of Community Health, Federal University of Health Sciences of Porto Alegre (UFCSPA), Porto Alegre, Brazil

**Keywords:** COVID-19, SARS-CoV-2 genomes, Severe acute respiratory syndrome coronavirus 2, Brazil, VUI-NP13L

## Abstract

In this study, we analyzed 340 whole genomes of SARS-CoV-2, which were sampled between April and November 2020 in 33 cities of Rio Grande do Sul, South Brazil. We demonstrated the circulation of two novel emergent lineages, VUI-NP13L and VUI-NP13L-like, and five major lineages that had already been assigned (B.1.1.33, B.1.1.28, P.2, B.1.91, B.1.195). P.2 and VUI-NP13L demonstrated a massive spread in October 2020. Constant and consistent genomic surveillance is crucial to identify newly emerging SARS-CoV-2 lineages in Brazil and to guide decision making in the Brazilian Public Healthcare System.

## Introduction

Since the emergence of SARS-CoV-2 (severe acute respiratory syndrome coronavirus 2) in China at the end of 2019, COVID-19 (coronavirus disease 2019) has been responsible for more than 3.8 million deaths worldwide until 21 June 2021 [1–3]. Brazil is second in terms of the number of COVID-19 deaths, only behind the USA, registering more than 500,000 deaths and 17,900,000 cumulative cases as of 21 June 2021 [4]. Rio Grande do Sul (RS), the most southern state of Brazil with a land area of 291,748 km^2^ and bordering Argentina and Uruguay, had its first COVID-19 case confirmed in early March 2020 [5]. More than one year later, RS reached a peak of infections leading to a collapse of the health system in March 2021 [6], with 29,672 deaths and 1,148,734 cases [7].

Even after one pandemic year and the introduction of SARS-CoV-2 vaccination worldwide, we have no forecasted end of the COVID-19 pandemic. Additionally, resurgence of COVID-19 after the first peak and after reaching high seroprevalence may drive positive selection of new lineages [8,9]. Constant epidemiological genomic surveillance through large-scale pathogen genome sequencing has played a major role in the detection and spatial–temporal distribution of SARS-CoV-2 lineages. Therefore, these data allow quasi-real-time tracking of viral dynamics around the globe [10–15].

Regardless of the volume of COVID-19 cases in Brazil, a task force created by the scientific community was able to sequence only approximately 0.03% of all positive SARS-CoV-2 cases through the pandemic’s first year [16,17]. Most of the Brazilian sequences from March and April 2020 belonged to two major clades of the B lineage (B.1.1), which were later named B.1.1.28 and B.1.1.33, and phylodynamic analysis indicated that they might have been seeded in the country by multiple events [18]. Resende et al. subsequently showed that the B.1.1.33 lineage was responsible for early community transmissions in Brazil [19]. Further studies revealed the emergence of two novel descendants of the B.1.1.28 lineage, P.1 (Amazonas) and P.2 (Rio de Janeiro), which were initially detected in the last quarter of 2020 and subsequently spread to all Brazilian regions [20,21]. The rapid upsurge of the P.1 lineage was particularly concerning, considering its higher transmissibility than the previous other Brazilian lineages [22].

Despite sequencing efforts, there is no accurate knowledge about the frequency of SARS-CoV-2 lineage distribution in Brazil, mainly due to sampling gaps in spatiotemporal strata and the low number of genomes [23,24]. This study aimed to reconstruct the spatiotemporal pattern of SARS-CoV-2 spread in the first year of the pandemic in South Brazil and reported the emergence of novel lineages. Whole-genome sequencing was performed for 340 SARS-CoV-2 genomes, the largest temporal (April and November 2020) monitoring of South Brazil to date.

## Materials and methods

### Bioethics, sample collection and processing

The study was approved by the Institutional Review Board of Moinhos de Vento Hospital under protocol number 32149620.9.0000.5330. The Clinical Epidemiology Laboratory (Epiclin), located in the Federal University of Health Sciences of Porto Alegre (UFCSPA) and supported by Moinhos de Vento Hospital, was responsible for conducting the diagnostic tests of SARS-CoV-2 in four regions belonging to the Northeast and the Metropolitan Mesoregions of the state of Rio Grande do Sul, southern Brazil. The samples sequenced in this study were collected in these four regions, covering a total of 33 municipalities described in Supplementary Data 1.

Diagnostic testing for SARS-CoV-2 using RT–PCR started in April 2020 and was completed in December of the same year. The total number of clinical samples received by the Epiclin Laboratory was 33,788 resulting in 31,318 individuals tested. Of the total tests carried out, 13,701 were positive for SARS-CoV-2 (40.55%).

Nasopharyngeal and oropharyngeal samples from SARS-CoV-2-infected individuals were collected according to the CDC guidelines [25]. Total nucleic acid was extracted from the samples using the MagNA Pure LC Total Nucleic Acid Kit – High Performance in the MagNA Pure Instrument (Roche) or MagMaxTM Viral/Pathogen (MVP III) Nucleic Acid Isolation kit (Applied Biosystems) in the KingFisher Flex System (Thermo Fisher Scientific). These samples were evaluated by the Allplex™ 2019-nCoV Assay (Seegene). Nucleic acid samples were immediately stored at −80°C.

### Study design

Total nucleic acids from positive samples of the Epiclin Laboratory diagnostic tests were selected considering a spatiotemporal outlook. Strata were defined using two criteria: epidemiological week and residence of the participant. The selected period includes epidemiological weeks between 17 (April 2020) and 49 (November 2020) from four regions (Novo Hamburgo, Taquara, Canoas, and Porto Alegre). In each of the 132 strata (33 weeks and 4 regions), samples were selected randomly for sequencing, totalling 353 RNA samples (Supplementary Methods).

### Library preparation and sequencing

Libraries were prepared using the QIASEQ SARS-CoV-2 Primer Panel (Qiagen) and QIAseq FX DNA Library CDI Kit (Qiagen) according to the manufacturer’s instructions, except for the annealing temperature of the primers. The QIASEQ SARS-CoV-2 Primer Panel contains a PCR primer set for whole-genome amplification of SARS-CoV-2 whose primer sequences were based on the ARTIC network nCoV-2019. To improve amplification of amplicon 64, the annealing temperature of primers was reduced by 1 (one) degree Celsius, from 65°C to 64°C (Supplementary Figure 1). The libraries were quantified using the Qubit^TM^ dsDNA HS Assay kit on a Qubit 4.0 fluorometer (Thermo Fisher Scientific) and normalized to equimolar concentrations. A pool of all of the normalized libraries was prepared and diluted to a final concentration of 10 pM and sequenced on the Illumina MiSeq platform using the MiSeq Reagent Kit v3 600 cycles (Illumina).

### Genome assembly

Raw paired-end reads were processed using a bioinformatic pipeline previously described with some modifications [26,27]. The reads were mapped with BWA 0.7.17 software [28] to the Wuhan-Hu-1 reference genome (NC_045512.2) and converted to BAM format using samtools v1.7 [29]. The primer sequences were trimmed with the iVar v.1.2.3 package, and consensus sequences were generated considering a Phred quality score minimum of 20 and N for regions with coverage depths less than 10 bases. The quality of the genome sequences was assessed using Nextclade version v. 0.14.1. Sequences classified as “bad” in the overall quality evaluation were discarded from further analyses. Viral genomes were deposited in GISAID, and the accession numbers are available in Supplementary Data 1 [28].

### Lineage identification, mutation analysis and phylogenomic analyses

Viral lineages were classified according to PANGOLIN (Phylogenetic Assignment of Named Global Outbreak LINeages) version 3.0.6 (pangoLEARN version 2021-06-05). Nucleotide and amino acid mutations were mapped using Nextclade version 0.14.1. Kernel density of the lineages during the epidemiological weeks was built using a script written in Python (Seaborn library) and the metadata of the Brazilian samples available in GISAID on 16 June 2021. covSPECTRUM (https://cov-spectrum.ethz.ch/) was utilized to check the prevalence of sets of mutations among the Pango lineages, using the Brazil dataset [30].

Phylogenomic analyses were carried out using Nextstrain, a suite of tools that includes subsampling, alignment, phylogenetic reconstruction, geographic and ancestral trait reconstruction, and inference of transmission events [31]. In the phylogenetic reconstruction, Nexstrain utilizes by default the software IQ-Tree and the substitution model GTR. For these analyses, our dataset of 340 local genome sequences was concatenated with the Nextstrain South America dataset (build 2021-03-03) (Supplementary Data 2). All 3,965 sequences were included in the pipeline (subsampling step was bypassed). Samples were classified according to their exposure location, taking into consideration the following rationale: RS; other regions of Brazil; South America, excluding Brazilian sequences; and other continents. Trait reconstruction was performed considering this custom geographic trait. An interactive phylodynamic and phylogenomic dataset of our study can be assessed in the link https://figshare.com/articles/dataset/auspice_final_11jun21_ed_zip/14781297. In order to visualize the Nextstrain build, the JSON file should be loaded at the following link: https://auspice.us/.

Novel lineages within the B.1.1.28 group were identified following the approach described in Rambaut et al., 2020. All genome sequences of the Pangolin B.1.1.28 lineage, recovered from the GISAID database (build 2021-05-31) (Supplementary Data 3), were concatenated with the B.1.1.28 genome sequences obtained in this study. Sequences were filtered using Augur Filter subcommand with a minimal length of 27,000 nucleotides, and subsequently aligned with MAFFT, FFT-NS-2 option with default parameters. The phylogenetic tree was built using IQ-Tree [32] using the GTR model and ultrafast bootstrapping with 1,000 replicates, and subsequently visualized in iTOL v6 [33]. The final tree was pruned using Newick Utilities [34], maintaining only sequences of the putative novel lineage and of the root (Wuhan sequences). A timetree inference and a “mugration” model between discrete geographic regions were conducted with Treetime version 0.8.1. Subsequently, it was plotted using a script written in R (ggtree library), colouring the branches according to the exposure location.

## Results

### Sampling, data acquisition and genome assembly

Between 24 April and 30 November 2020, 13,701 nasopharyngeal swab samples from four regions of Rio Grande do Sul were laboratory-confirmed to have SARS-CoV-2 infection at Epiclin Laboratory. From those, 353 samples were selected by region and time for whole-genome sequencing. We verified that only seven of 96 consensus sequences of the first sequencing run presented a complete region spanning the position 19204–19616, corresponding to amplicon 64 of the ARTIC framework (Supplementary Figure 1). For subsequent sequencing runs, the depth of the amplicon 64 region was increased by lowering the annealing temperature of the multiplex tiling-PCR by 1°C.

After sequencing, 340 genomes displayed the minimum quality requirements for further analyses. The number of paired-end reads generated per sample varied from 132,396 to 3,480,771. Genome sequences comprised at least 97.44% of the Wuhan-Hu-1 reference genome (GenBank accession number: NC_045512.2), independent of the threshold cycle (Ct), with depth coverage ranging from 554x to 14,935x (Supplementary Data 1). These results are associated with the optimization of the sequencing library preparation protocol (Supplementary Figure 1).

Our sequencing effort was composed of a homogeneous distribution of samples along a temporal window from April to November 2020, when compared with a dataset available for the same period (Supplementary Figure 2). Our study more than doubled the number of SARS-CoV-2 sequences from Rio Grande do Sul of this timeframe in GISAID (from 294 to 634, GISAID on 15 June 2021).

### Monitoring of SARS-CoV-2 lineages in South Brazil

**Figure 1.**
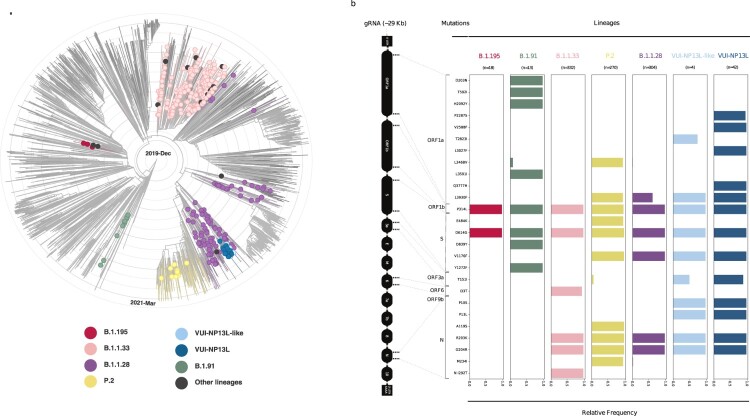
Phylogenomic reconstruction of the SARS-CoV-2 genome. (a) Phylogenetic tree of 340 sequences described in this study combined with a dataset of 3,625 South American genomes. Branch lengths are proportional to the collection date (between Abril 2020 and March 2021). Concentric circular rings represent the timeline (interval every three months). Each filled circle represents our SARS-CoV-2 sequences, and they are coloured according to lineage (legend box). (b) Highlighter plot showing SARS-CoV-2 mutation patterns along the genome evaluated here. Mutations are colour-coded according to the lineages shown in Figure 1A legend.

Genomes reported in this study were assigned to 14 lineages based on the proposed dynamic nomenclature of PANGOLIN [35,36] (Supplementary Data 1). To determine the phylogenomic and epidemiologic characteristics of the SARS-CoV-2 genomes, we performed a phylogenomic analysis containing the genomes described in our study combined with a dataset of 3,625 global representative genomes, enriched for South American sequences (1,340 Brazilian genomes; Supplementary Data 2). The lineages were distributed in five major groups: B.1.1.28 (*n* = 117; 27.9%), P.2 (*n* = 14; 4.1%), B.1.1.33 (*n* = 183; 53.8%), B.1.91 (*n* = 9; 2.6%), and B.1.195 (*n* = 3; 0.9%) (Figure 1a). Fourteen genomes (2.4%) belonged to low prevalence lineages and/or did not cluster within the predicted lineages. These lineages were identified as “others”: B.1 (*n* = 8), B.1.1 (*n* = 1), B.1.1.332 (*n* = 1), B.1.1.70 (*n* = 1), B.1.1.277 (*n* = 1), B.1.212 (*n* = 1), N.1 (*n* = 1).

Among all genomes sequenced in this study, we found a total of 503 non-synonymous mutations compared to the reference Wuhan-Hu-1 sequence. Non-synonymous mutations were found mainly in ORF1a (211 mutations), ORF1b (91), S (60), ORF3a (39), N (37), ORF8 (24), ORF7a (17), ORF9b (8), M (7), ORF6 (4), E (3), and ORF7b (2) (Supplementary Figure 3).

It is worth noting that B.1.91 presents two amino acid changes in the spike protein (D614G and D839Y) (Figure 1b). In turn, all P.2 samples have the E484K, V1176F, and D614G mutations in the spike protein (Figure 1b), and most of those from RS present the synonymous mutation T3766C in ORF1a gene (Supplementary Figure 4).

Within the cluster B.1.1.28, 22 genomes presenting the amino acid substitution N P13L divided into two groups, VUI-NP13L and VUI-NP13L-like (Figure 1a). A thorough analysis showed that, besides the N P13L, the amino acid substitutions ORF3a T151I and ORF9b P10S are frequent in these lineages (Figure 1b). According to covSPECTRUM, this set of non-synonymous mutations, N P13L, ORF3a T151I and ORF9b P10S, are exclusively found in sequences classified as B.1.1.28 in the Brazil dataset. Concerning the VUI-NP13L lineage, we found additional four exclusive amino acid substitutions in ORF1a, P2287S, V2588F, L3027F and Q3777H in relation to VUI-NP13L-like.

**Figure 2.**
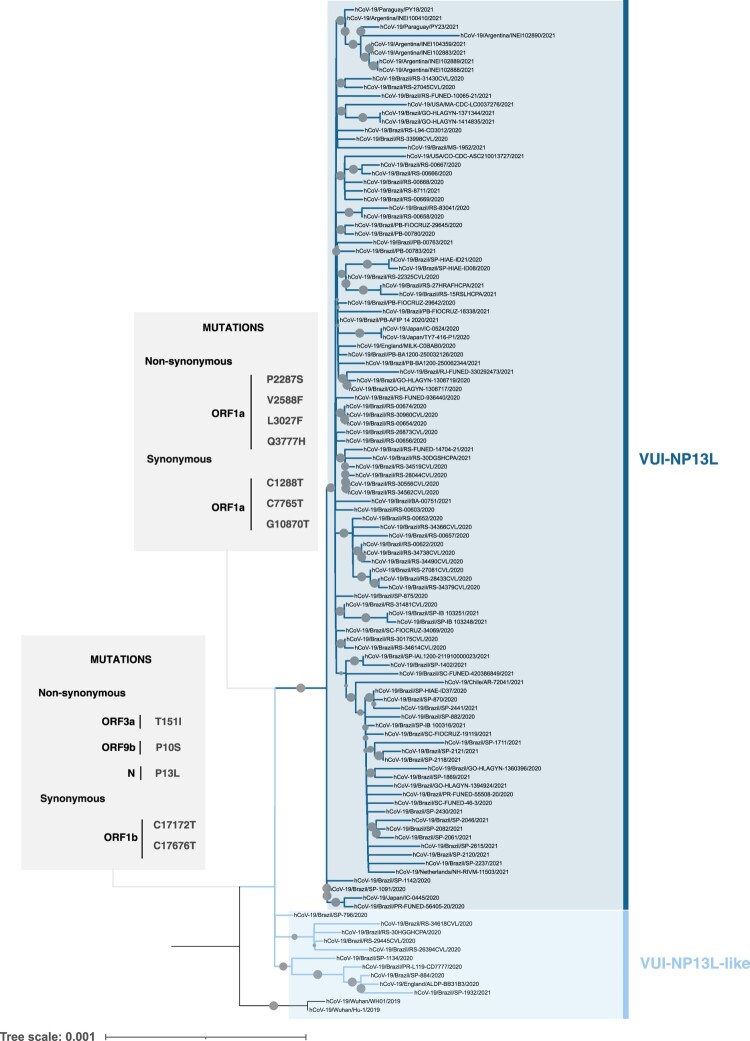
Phylogenomic tree of VUI-P13L-like and VUI-P13L lineages. Phylogenetic inference was performed using all B.1.1.28 sequences available in GISAID. The cluster comprising VUI-P13L-like and VUI-P13L was evident. Mutations shared between VUI-P13L-like and VUI-P13L (in ORFa, ORF9b and N) as well as unique amino acid changes and synonymous mutations to VUI-P13L present in ORF1a are highlighted in the boxes. Circles positioned over branches represent ultrafast bootstrap values, and their diameters are proportional to the range from 70 to 100.

The VUI-NP13L and VUI-NP13L-like lineages were then validated in the context of all B.1.1.28 genome sequences available in GISAID (Supplementary Figure 5; Supplementary Data 3). Again, a discrete group of 115 genome sequences presenting the NP13L amino acid signature was formed (Figure 2) that subdivides into two minor clusters presenting the characteristic amino acid signatures found previously (Figure 2, Supplementary Figure 6a). We found that most of the sequences from both subgroups also share two synonymous mutations in the ORF1b gene, C17172T and C17676T (Figure 2, Supplementary Figure 6(b)). The VUI-NP13L lineage in turn presents additional three synonymous mutations in the ORF1a gene (C1288T, C7765T and G10870T) (Figure 2, Supplementary Figure 6b).

### Temporal and spatial dynamics of SARS-CoV-2 lineages

Based on the phylogenomic analysis (Auspice file available in https://figshare.com/articles/dataset/auspice_final_11jun21_ed_zip/14781297) and the PANGO lineage description list (https://cov-lineages.org/lineage_description_list.html), most of the genomes are derived from Brazilian lineages: B.1.1.33, B.1.1.28 and P.2. In addition, non-brazilian lineages, B.1.91 and B.1.195, were identified (*n* = 12). The B.1.91 group is immersed within an European clade, while the B.1.195 group is composed of sequences recovered from different countries, mainly from South America.

**Figure 3.**
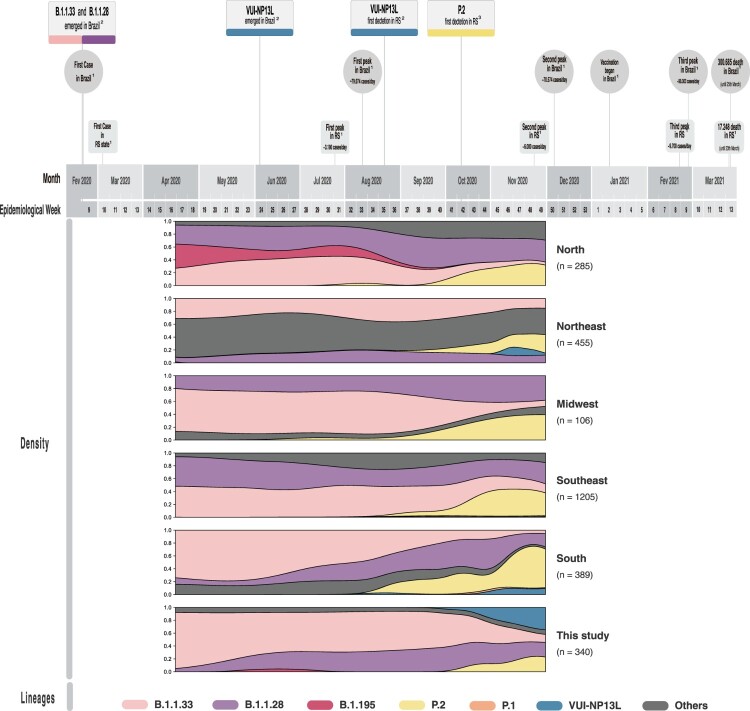
Timeline and density plot of Brazilian SARS-CoV-2 genomes during epidemiologic weeks. The timeline of the main events and circulating lineages of the SARS-CoV-2 pandemic in RS and Brazil from epidemiological weeks 9 of 2020 to 13 of 2021 are highlighted, representing the first year of the pandemic. The frequency of SARS-CoV-2 lineages found in this study in comparison to lineage distribution in other Brazilian regions is shown during our sampling period. (1) Data available in https://covid.saude.gov.br/; (2) Origin dates inferred in this study; (3) GISAID.

The profile of SARS-CoV-2 epidemiological progression over time highlighting main events in the context of Brazil and RS epidemics is shown in Figure 3. At the beginning of the sampled period (April), B.1.1.28 and B.1.1.33 were the predominant lineages circulating in our samples as well as in other Brazilian regions.

Regarding the P.2 lineage, it was initially detected in Rio de Janeiro (Brazil/RJ-FIOCRUZ-2188-1P/2020), Southeast Brazil, and Ceará (hCoV-19/Brazil/CE-IEC-177339/2020), Northeast Brazil, on 13 and 15 April 2020, respectively (epidemiological week 16). Although the first sample of the P.2 lineage in RS was recovered in Porto Alegre from 31 August 2020 (Brazil/RS-L93-CD3010/2020), the following samples were only collected from October 2020 onwards. In our dataset, the oldest P.2 sample is from Porto Alegre, recovered on 22 October 2020 (epidemiological week 43). The increase of P.2 lineage prevalence began earlier in other Brazilian regions than in our study (Figure 3).

The VUI-NP13L lineage was first detected on 31 July 2020 in São Paulo in a patient from Goiás (Brazil/SP-HIAE-ID37/2020), and then detected in São Paulo (Brazil/SP-1091/2020) on 8 August 2020. In the RS, VUI-NP13L was first detected in Porto Alegre on 29 August 2020 (Brazil/RS-L94-CD3012/2020). Our study revealed the second detection of this lineage, a sample obtained in Taquara on 7 October 2020 (epidemiological week 41). Since then, our findings have shown a massive rise of this lineage (Figure 3).

Based on our phylodynamic analysis, it was possible to infer that VUI-NP13L emerged in June 2020 in Southeast Brazil (Supplementary Figure 7). Phylogeographic analysis indicated that after spreading in the RS, the VUI-NP13L lineage further arrived in Northeast Brazil, North America, Asia, Europe and South America, being recently found in Argentina (9 April 2021) (Supplementary Figure 7).

**Figure 4.**
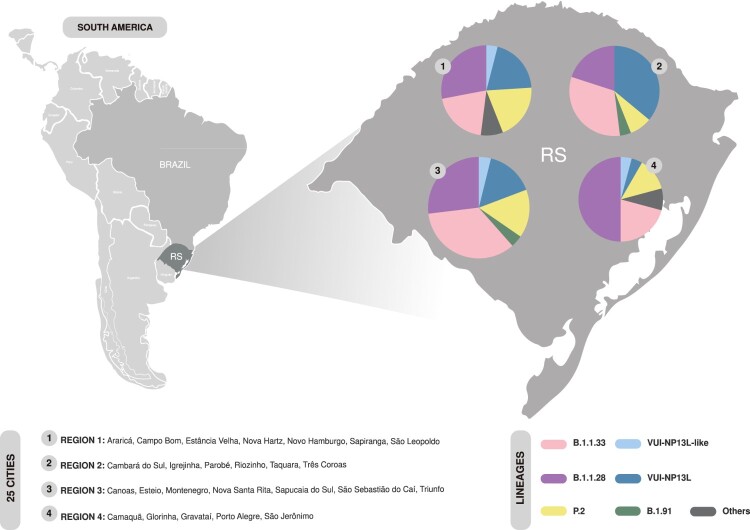
Spatial distribution of the SARS-CoV-2 lineages between 7 October and 30 November 2020 (epidemiological weeks 41–49) in four regions of RS. The pie charts represent lineage composition among the different RS regions. The diameter of each pie chart is proportional to the number of sequences belonging to each sampled region.

The geographic distribution of lineages was evaluated in four RS-sampled regions since the first detection of VUI-NP13L in our sampling (Figure 4). B.1.1.28, B.1.1.33 and VUI-NP13L were found across all regions, while the B.1.91 lineage was found only in regions 3 and 2, displaying a frequency of approximately 4%. We detected P.2 and VUI-NP13L lineages distributed in 8 and 11 cities, respectively. VUI-NP13L was the most prevalent lineage circulating in region 2 (36%) and represented 15.4 and 20% of genomes in regions 3 and 1, respectively. On the other hand, P.2 was more frequent than VUI-NP13L in region 4, but equally frequent in regions 3 and 1.

## Discussion

This is the one of the largest genomic studies of SARS-CoV-2 in Brazil considering the number of sequences and the temporal monitoring window. We characterized two new lineages, VUI-NP13L and VUI-NP13L-like, and identified the massive spread of P.2 and VUI-NP13L lineages since October 2020 in RS.

The main strength of our study was the analysis of a substantial number of genomes, which represent a geographic region composed of 33 cities over 8 months. This approach was decisive to evaluate the lineage prevalence across sampled regions and monitor the emergence of new variants of interest. We optimized the sequencing library preparation protocol, improving SARS-CoV-2 genome amplification and enhancing sequencing performance. Although we investigated different regions of the RS, including the metropolitan region, the sampling was only carried out in the most southernmost state of Brazil, which presents different culture, climate, geographic accesses and population’s genetic composition in relation to other Brazilian states. Therefore, we cannot extrapolate our findings to the rest of Brazil, a country of continental dimensions. Genomes sequenced in our study are restricted to 2020; however, we verified that the VUI-NP13L and P.2 lineages are still active across Brazil in 2021.

Of the five main circulating lineages, B.1.1.33 and B.1.1.28 were the most predominant, mainly in the initial period of the study. Our findings are supported by other Brazilian sequencing studies, which showed that B.1.1.33 and B.1.1.28 are the most prevalent lineages of the country, including South Brazil [16].

Regarding phylogeographic analyses, B.1.1.33 and B.1.1.28 lineages were imported from other Brazilian states, and subsequently transmitted among the local community. According to Resende et al. [19], the B.1.1.33 lineage was first detected in São Paulo, Southeast Brazil, on 9 March 2020 and then disseminated across the country [16,24,37,38]. This lineage was also associated with outbreaks in Argentina and Uruguay, countries bordering RS [38,39].

Our phylogenomic analyses provided evidence that B.1.1.28 is a paraphyletic group composed of several subclades of distinct evolutionary origins and unique genetic signatures. Therefore, we found that the B.1.1.28 lineage may have arrived in RS during multiple episodes, although Francisco Jr. et al. suggested that it was introduced by a single seeding event [17]. Despite most lineages having been introduced from other Brazilian states, it seems that B.1.91 was directly imported from Europe. However, it is worth noting that for reliable geographic transmission inference, careful sampling is necessary. Available genomes in GISAID are unbalanced among different Brazilian regions, which may lead to incorrect inferences about virus origins. Furthermore, phylodynamic modelling depends on reliable phylogenetic inferences, being the latter difficult to be established given the large number of SARS-CoV-2 sequences with low diversity [40].

Our results also indicated a sharp increase in the P.2 lineage almost concurrently with VUI-NP13L. Recently, Lamarca et al. (2021) reported P.2 as the most abundant lineage in Northeast and Southeast Brazil. Furthermore, the authors revealed the beginning of its circulation in March 2020, exhibiting an intense transmission between December 2020 and January 2021 [37].

Precise detection of the origins of an outbreak succeeds only when the genetic background of the pathogen is known. In this sense, our dataset was crucial for tracing the beginnings of an outbreak in the RS caused by the VUI-NP13L lineage.

The VUI-NP13L was first identified as a potential novel lineage by Francisco Jr. et al. (2021) [17], and they named it according to the P13L amino acid change in the N protein. However, in this study we demonstrated two viral sublineages possessing this modification, VUI-NP13L and VUI-NP13L-like, and described their genetic signatures. We consider that the VUI-NP13L lineage should be named according to the criteria described by Rambault et al. [35,41]. Although we had submitted a lineage proposal on 9 March 2021 (https://github.com/cov-lineages/pango-designation/issues/29), at the moment of this writing, it is still under consideration.

VUI-NP13L probably emerged in Southeast Brazil around June 2020, and it was identified in South Brazil in August 2020. According to an independent phylodynamic reconstruction, VUI-NP13L rapidly arrived in the southeastern and northeastern regions of Brazil and seems to have been exported to Argentina, Paraguay, Japan, the Netherlands and England [37]. The success of the spread of VUI-NP13L, beyond having important mutations in the spike protein (V1176F, D614G), also shared by P.2 and P.1 lineages and inherited from B.1.1.28, could be justified by its unique amino acid changes [16,42,43].

The analysis of the mutational signatures showed that VUI-NP13L and VUI-NP13L-like share three non-synonymous mutations: ORF3a (T151I), ORF9b (P10S) and N (P13L). The ORF3a is implicated with virulence, infectivity and virus release, and its T151I mutation is located in a cysteine-rich domain (positions 81–160), which forms interchain disulfide bonds on the interior side of the viral envelope with the spike protein [44,45]. Since the ORF9b is involved in the suppression of IFN-I antiviral response [46], further investigation is necessary to evaluate the role of P10S mutation on this process. In respect to the N protein, the P13L mutation is related to lower mortality rates of SARS-CoV-2 [46,47].

Regarding VUI-NP13L, we determined a signature of four amino acid changes in ORF1a. This gene is known to encode a polyprotein involved in the replication complex [48]. Previous studies concerning SARS-CoV and MERS indicated the role of ORF1a in survival and adaptation to the host [49,50]. Further, Forni et al. (2016) suggested that ORF1a positive selection might contribute to host shifts or immune evasion [49]. According to them, adaptive evolution is unevenly distributed in the ORF1a proteins, but is mainly ongoing at the protein NSP3, a papain-like proteinase that acts on the release of essential proteins for viral activity [51]. The VUI-NP13L lineage possesses two mutations at this protein, P2287S and V2588F. The L3027F mutation was found in the NSP4, a protein that has an essential role in the viral replication and membrane rearrangement [52]. Lastly, NSP6 protein, which prevents the degradation of viral components by the lysosomes [53], presented the Q3777H mutation. The balance between the different mutational contributions on fitness may impact the ability of the lineage to persist. However, we cannot discard the increase in frequency of VUI-NP13L due to random causes such as a “founder effect” [43].

Our study reinforces the importance of consistent and continuous genomic surveillance for evaluating the genomic background of SARS-CoV-2 in a given spatiotemporal setup. These data are fundamental for inferring SARS-CoV-2 outbreaks and revealing signatures, activity and origins of the lineages. Furthermore, genome surveillance is an invaluable resource to guide decision making of the Brazilian Public Healthcare System. Immunization campaigns could be especially affected by the emergence of novel lineages that evade antibodies generated by current vaccines. Future studies are needed to assess the fate of P.2 and VUI-NP13L over time, and if they are still observed, we need to evaluate the impact of amino acid changes on the fitness of these lineages.

## Supplementary Material

supp_data_4_acknowlegments_21jun21.xlsx

supp_data_3_b1128_21jun21.xlsx

supp_data_2_south_america.xlsx

supp_data_1_ed_final_10jun21.xlsx

supp_information_p41_2021_21jun21.docx
