# Supplementary material for: Emergence of the novel SARS-CoV-2 lineage VUI-NP13L and massive spread of P.2 in South Brazil
Source: Emerg Microbes Infect. 2021 Jul 15;10(1):1431–40. doi: 10.1080/22221751.2021.1949948 (PMC8284128; doi:10.1080/22221751.2021.1949948)
Supplement: supp_information_p41_2021_21jun21.docx [file TEMI_A_1949948_SM7921.docx]

**Supplementary Information**

**Title: Emergence of the novel SARS-CoV-2 lineage P.4.1 and massive spread of P.2 in South Brazil**

Fernando Hayashi Sant’Anna^1^, Ana Paula Muterle Varela^2^, Janira Prichula^2^, Juliana Comerlato^1^, Carolina Baldisserotto Comerlato^1^, Vinicius Serafini Roglio^1^, Gerson Pereira de Almeida^3^, Flavia Moreno^3^, Adriana Seixas^2^, Eliana Márcia Wendland^1,4*^

^1^Hospital Moinhos de Vento, PROADI – SUS, Porto Alegre, Brazil

^2^Graduate Program in Biosciences, Federal University of Health Sciences of Porto Alegre (UFCSPA), Porto Alegre, Brazil

^3^Department of Chronic Conditions and Sexually Transmitted Infections, Ministry of Health, Brasília, Brazil.

^4^Department of Community Health, Federal University of Health Sciences of Porto Alegre (UFCSPA), Porto Alegre, Brazil

* e-mail: elianawend@gmail.com

Supplementary Data 1. **Metadata of the** **340 whole-genome sequences obtained in this study.**

Supplementary Data 2. **South America dataset of SARS-CoV-2 genomes (n = 3,625) retrieved from GISAID** **enriched for South America and used as a reference for the construction of the phylogeny, as well as their respective accession numbers and corresponding lineages.**

Supplementary Data 3. **Dataset of B.1.1.28 sequences from GISAID used for phylogenomic analysis.**

Supplementary Data 4. **Acknowledgement table.**


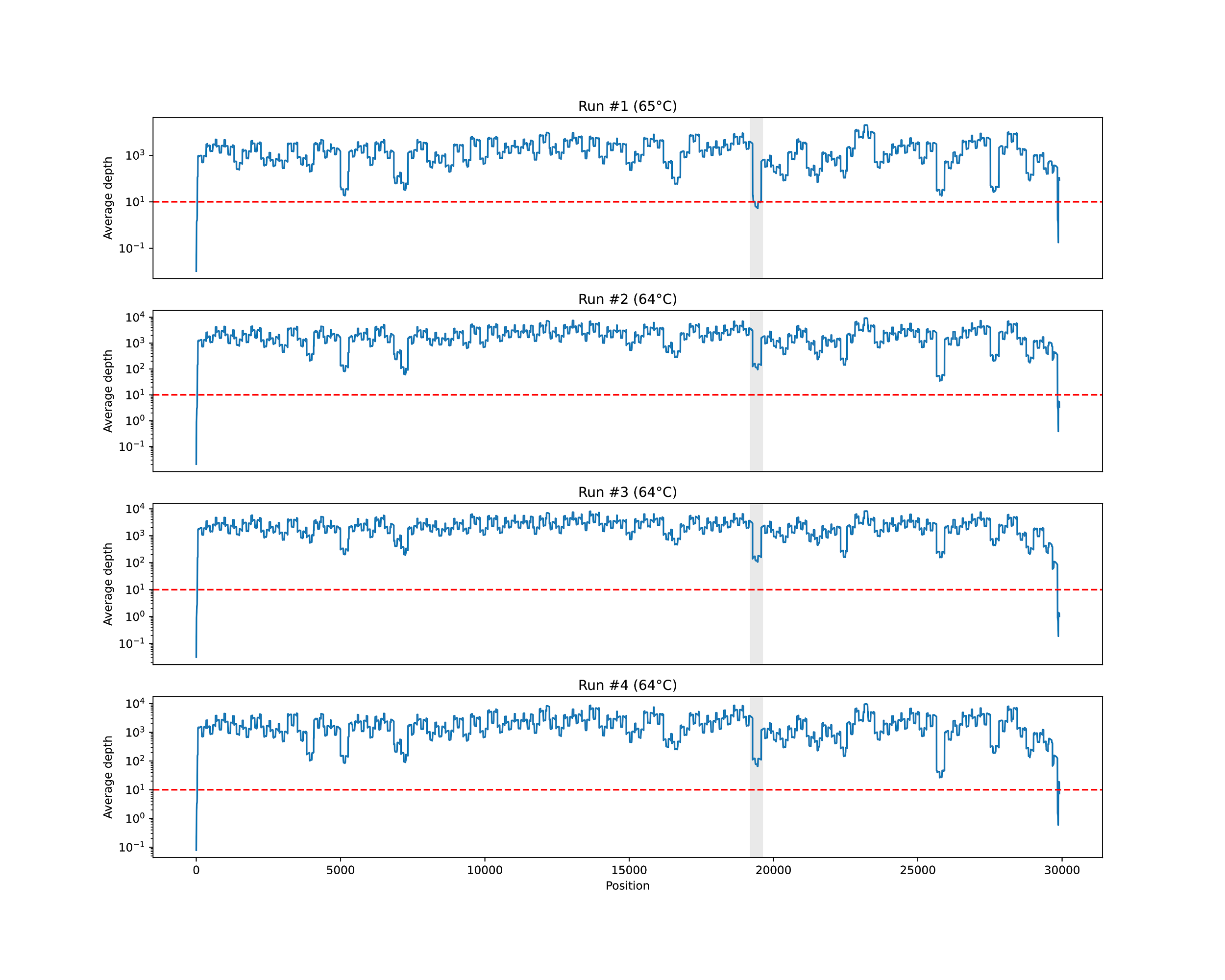


Supplementary Fig. 1. **SARS-CoV-2 genome amplification performance by target position using two different annealing temperatures.** Plots of the depth coverage of the SARS-CoV-2 genomes per position in the different sequencing runs. The first plot represents the mapped genome profile with annealing temperature at 65 °C in the multiplex tiling-PCR, and the other three show the profile at 64 °C. The gray stripe highlights the region spanning the position 19204 to 19616, corresponding to amplicon 64 of the ARTIC framework.

**
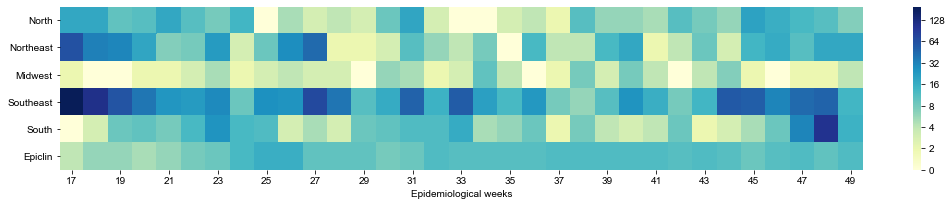
**

Supplementary Fig. 2. **Distribution of SARS-CoV-2 genomes in Brazilian regions and Epiclin from April to November (epidemiological weeks 17 to 49).** Heatmap showing SARS-CoV-2 genomes in the five Brazilian regions compared to the samples sequenced in this study between epidemiological weeks 17 and 49. Brazilian regions are in the rows and epidemiological weeks are in the columns. The relative abundance is represented by colors (white, lowest abundance; dark blue, highest abundance), as indicated in the legend. Average number and the standard deviation of genomes per week: North, 8.64 ± 5.82; Northeast, 13.78 ± 13.86; Midwest, 3.21 ± 2.46; Southeast, 36.52 ± 36.92; South, 11.79 ± 18.35; Epiclin, 10.30 ± 2.74.

**
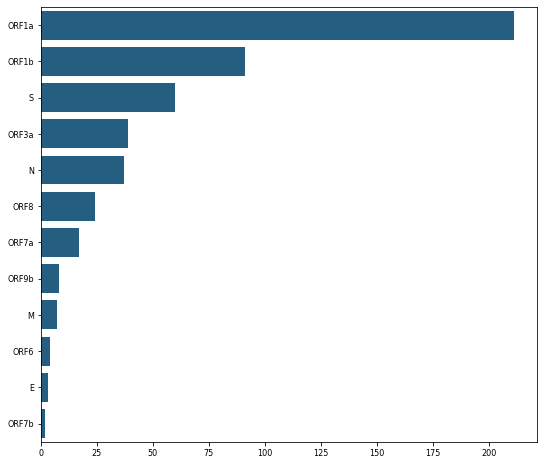
**

Supplementary Fig. 3. **Frequency of amino acid changes of the proteins of the SARS-CoV-2 samples sequenced in this study.**


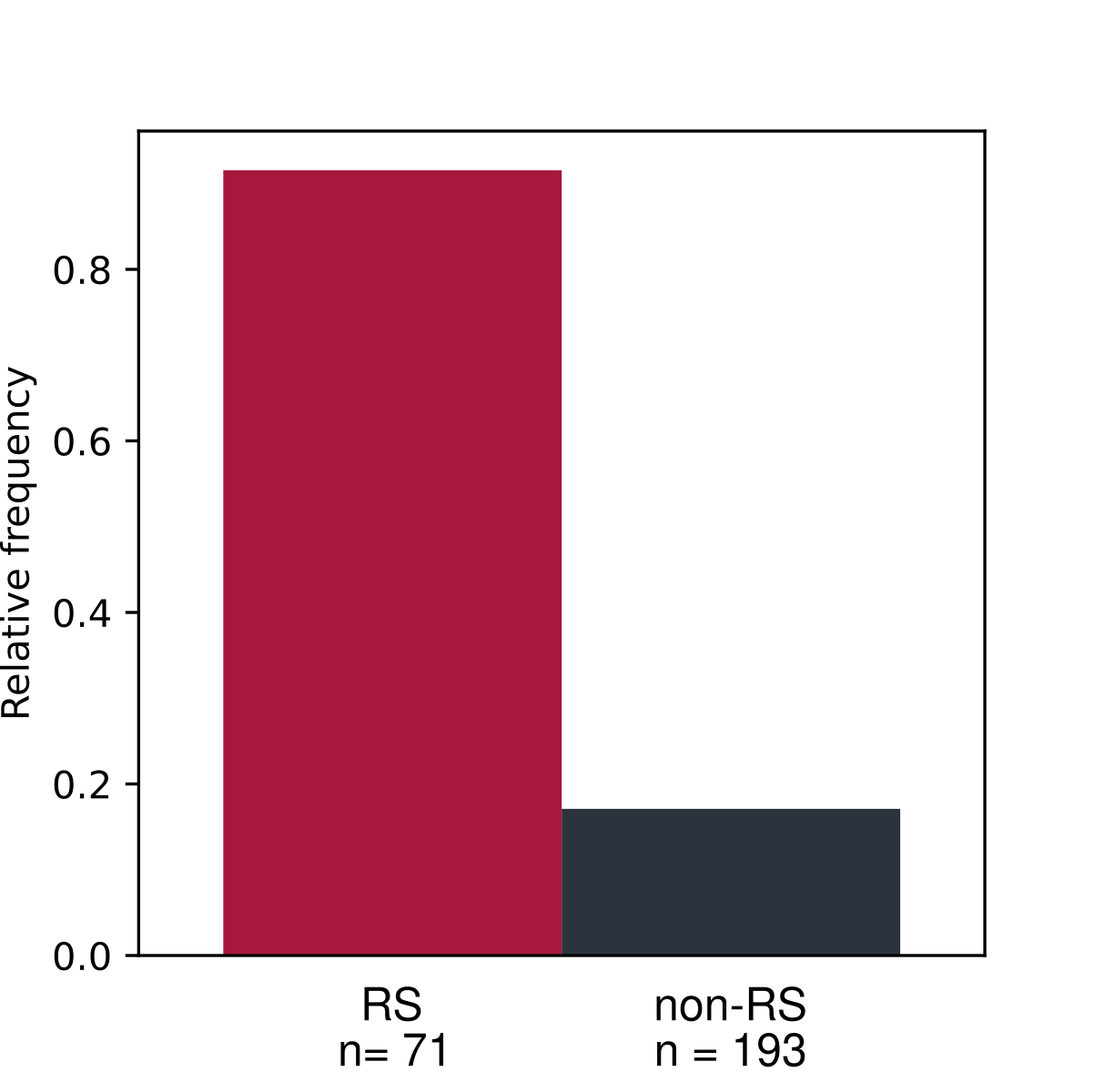


Supplementary Fig. 4. **Comparison of the prevalence of the synonymous mutation T3766C between P.2 samples from RS and the rest of Brazil.**


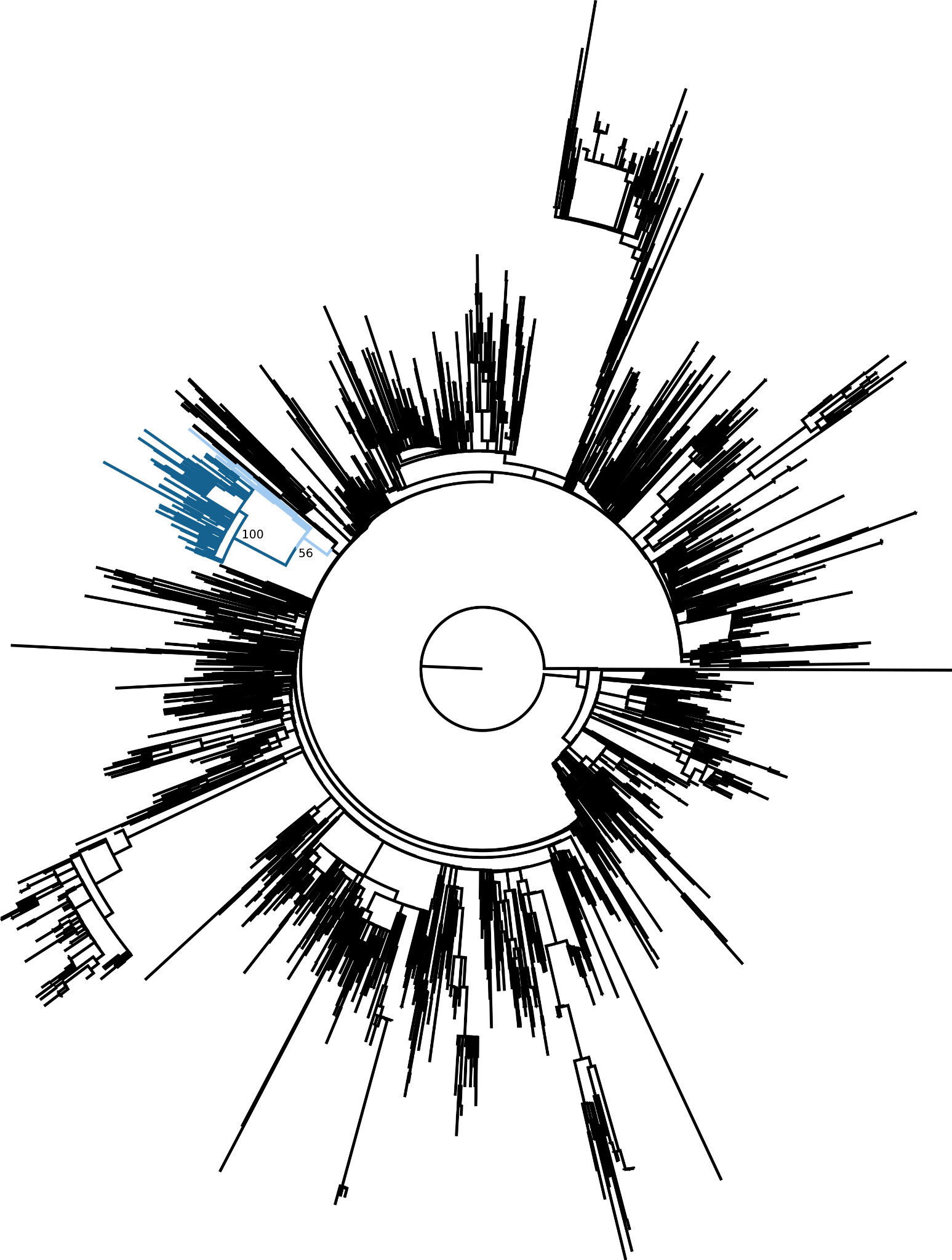


Supplementary Fig. 5. **Phylogenomic reconstruction of the SARS-CoV-2 B.1.1.28 lineage available in GISAID.** Clusters of novel lineages, VUI-NP13L and VUI-NP13L-like, are shown in dark and light blue colors, and other sequences of the B.1.1.28 lineage are shown in black. The ultrafast bootstrap values of the clades of interest are shown next to the nodes. The phylogeny was estimated with 1,000 bootstrap replicates.


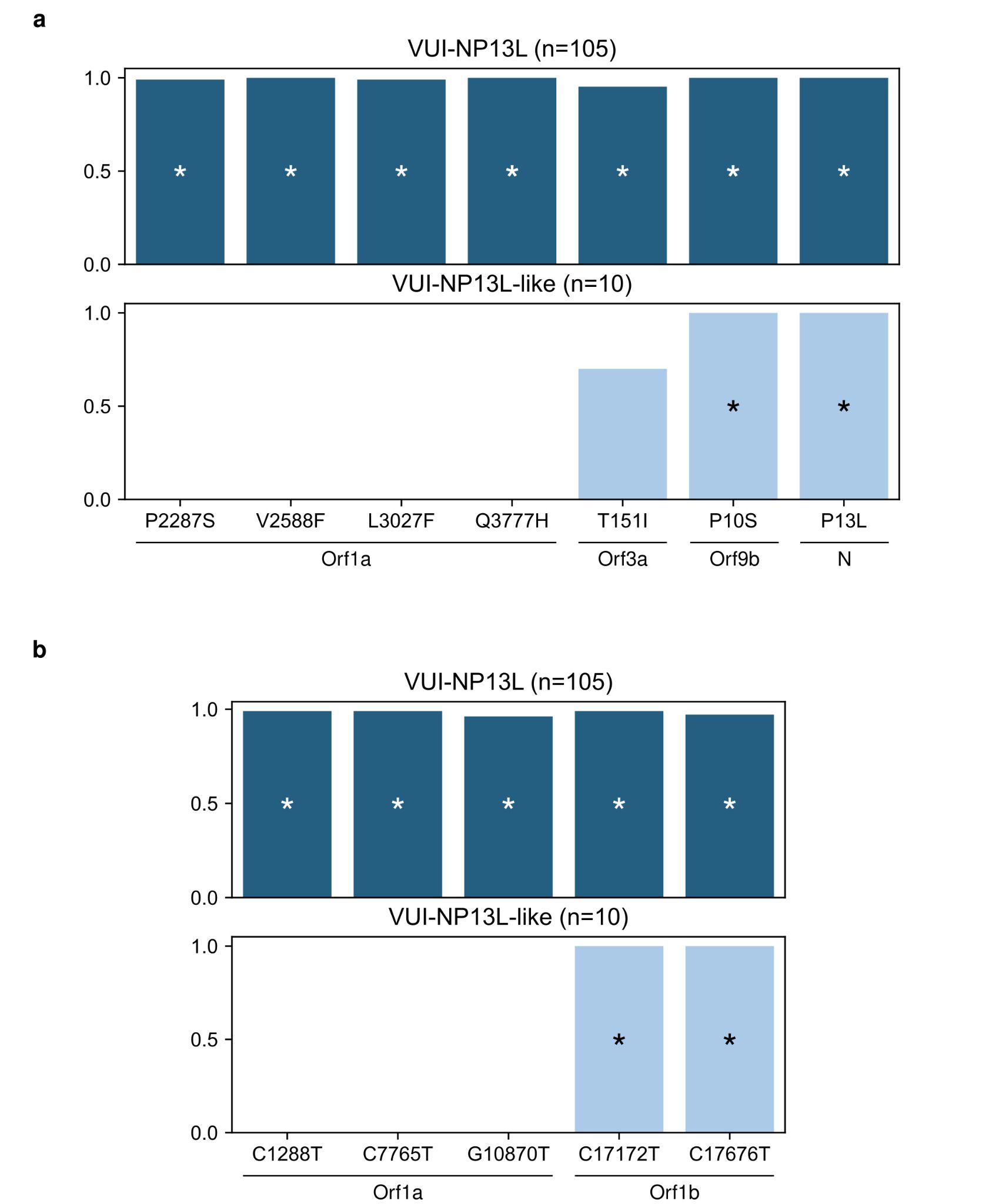


Supplementary Fig. 6. **Mutational signatures of the VUI-NP13L and VUI-NP13L-like lineages.** Mutations presenting a prevalence of at least 95% are marked with an asterisk (*). **a** Only the non-synonymous mutation Orf3a T15L presented a prevalence of 70%. VUI-NP13L contains seven non-synonymous mutations in four SARS-CoV-2 genes and VUI-NP13L-like shows three mutations in three genes. **b** Regarding synonymous mutations, five were found in the VUI-NP13L strain in the Orf1a and Orf1b genes, while the VUI-NP13L-like strain showed only two mutations in the Orf1b gene.


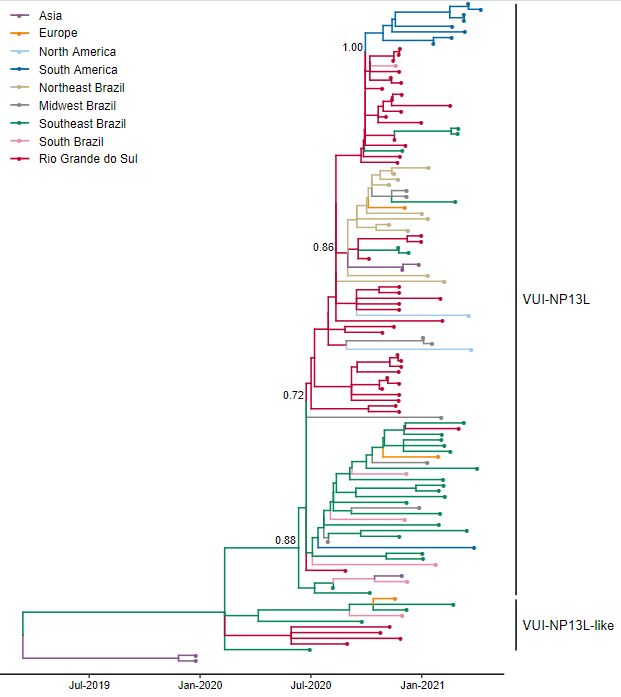


Supplementary Fig. 7. **Phylogeographic analysis of the VUI-NP13L lineage.** Time-scaled tree of all the SARS-CoV-2 VUI-NP13L and VUI-NP13L-like samples found in GISAID. Terminal branches are colored according to the exposure location of the sample. Internal branches are colored according to the most probable location of the LCA of the clade. The inferred probabilities of finding an internal node in a particular location of the main seeding events are shown next to the nodes.

**Supplementary Methods**

**Sampling criteria**

The samples sequenced in this study are from four main regions named regions 1 to 4: Novo Hamburgo (region 1), Taquara (region 2), Canoas (region 3), and Porto Alegre (region 4). These regions are located in the State of Rio Grande do Sul in southern Brazil. Each region comprises following cities: Novo Hamburgo covered 9 cities (Araricá, Campo Bom, Dois Irmãos, Estância Velha, Nova Hartz, Novo Hamburgo, Portão, São Leopoldo and Sapiranga); Taquara included 7 cities (Cambará do Sul, Igrejinha, Parobé, Riozinho, Rolante, Taquara and Três Coroas); Canoas included 10 cities (Canoas, Esteio, Harmonia, Montenegro, Nova Santa Rita, Pareci Novo, São Sebastião do Caí, Sapucaia do Sul and Triunfo, Vera Cruz); and Porto Alegre included 7 cities (Porto Alegre, Gravataí, Cachoeirinha, Camaquã, Alvorada, São Jerônimo, Glorinha), total 33 cities that represent the northeast and the metropolitan region of the State.
